# Efficient evaluation of the Open QC task fMRI dataset

**DOI:** 10.3389/fnimg.2023.1070274

**Published:** 2023-02-17

**Authors:** Joset A. Etzel

**Affiliations:** Cognitive Control and Psychopathology Laboratory, Department of Psychological and Brain Sciences, Washington University in St. Louis, Saint Louis, MO, United States

**Keywords:** fMRI, Quality Control, human, R, task

## Abstract

This article is an evaluation of the task dataset as part of the Demonstrating Quality Control (QC) Procedures in fMRI (FMRI Open QC Project) methodological research topic. The quality of both the task and fMRI aspects of the dataset are summarized in concise reports created with R, AFNI, and knitr. The reports and underlying tests are designed to highlight potential issues, are pdf files for easy archiving, and require relatively little experience to use and adapt. This article is accompanied by both the compiled reports and the source code and explanation necessary to use them.

## 1. Introduction

This article is part of the Demonstrating Quality Control (QC) Procedures in fMRI (FMRI Open QC Project) methodological research project, and describes procedures for efficiently evaluating its task dataset. These procedures examine both the task (behavioral performance, stimuli presentation, etc.) and fMRI (motion, appearance of preprocessed images, etc.) aspects of the dataset. The code and criteria presented here are versions of that used for the Dual Mechanisms of Cognitive Control (DMCC; Braver et al., 2021; Etzel et al., 2022) and multiple other projects in the Cognitive Control and Psychopathology Laboratory at Washington University in St. Louis (USA), and we hope will be useful and easily adapted by others.

QC procedures are often a balancing act between being so cursory that important problems are not identified, and so onerous that QC procedures are skipped entirely. The files and procedures presented here attempt to thread the needle; clearly highlighting the problems of greatest potential risk to the dataset and analysis integrity and validity, while remaining concise and easy to learn. These are intended to serve as a first step; a QC summary to allow efficient screening for potential issues, not to include all the details necessary to investigate any issues found.

These procedures are built around two dynamic report documents edited for the Open QC task fMRI dataset. The dynamic report framework is particularly well-suited to scientific programming because images, results, source code, and discussion are together in a single document. These reports are compiled to pdf files (convenient for archiving and have the same appearance wherever viewed), but there are many options for both output format and programming language. Regardless of the implementation details, I urge scientists to strive for clarity, simplicity, and stability when writing QC (or analysis) code over brevity and style purity, and hope that the documents included here can serve as a useful template.

## 2. Methods

One of the few statements a group of fMRI methods experts might all agree with is that there is a wide variety of methods for fMRI acquisition, processing, and analysis, none of which are unequivocally “best” for all (or even a specific) research questions. Given this methodological variety, quality judgments also widely diverge; the same images may be deemed suitable for one analysis, but too flawed for another. There is also lack of consensus on which images to evaluate for quality, with some researchers using the raw images, others the preprocessed, and yet others a combination or after a processing pipeline used only for QC. Thus, one of the first decisions when approaching a new fMRI project is to determine which QC aspects are most relevant for the study, and how they will be evaluated.

In general, I believe the QC procedures should be dictated by each project's hypotheses and analyses, not by a standard protocol or fixed thresholds. Accordingly, I suggest performing QC on the images preprocessed as they will be for analysis. For example, if surface analyses are planned, the QC should include the surface reconstruction, and temporal mean, standard deviation (sd), and tSNR (temporal signal to noise ratio, here, mean/sd) images of the vertex timecourses (rather than the voxels used here). If a particular software package has been chosen, then the QC should be done using the images and motion parameters created by that package. Similarly, if images will be analyzed in subject space, the QC should be in subject space as well.

The reasoning behind this suggestion to perform QC on the preprocessed images is as a minimum, essential step; not to preclude other tests, but to maximize the likelihood of detecting an error arising anywhere in the pipeline. If a preprocessed image has high quality, its raw version is likely also of high quality, but a poor preprocessed image may or may not be the result of a low-quality raw image (e.g., if the participant moved during field mapping, warping may be introduced during the distortion correction preprocessing step). Again, I am not advising against including additional QC steps; procedures like evaluating image registration may be critically important in some cases. But I do advise that the preprocessed images always be examined for quality; that other image QC steps be in addition, not a replacement.

When considering task fMRI QC, participant behavior (e.g., task performance) is also of fundamental importance. Note that this is not evaluating whether the participant responded as theoretically predicted, but rather confirming that they were attempting to perform the instructed task (and not, say, sleeping or responding randomly). If the task requires frequent responses (e.g., button push and spoken word), response frequency may be useful as a proxy for attentiveness: long stretches without a response suggests the participant stopped performing the task. Other tasks may not require responses during the imaging session, but rely upon something like monitoring eye gaze or the results of a memory test performed after the session. Whatever the paradigm, for QC the aim is to determine a non-biased way to identify participants who did not have the minimally-valid task performance.

### 2.1. Data processing

The FMRI Open QC Project task dataset (Gorgolewski et al., 2017; Markiewicz et al., 2021) was provided in BIDS (Brain Imaging Data Structure; Gorgolewski et al., 2016) format specifically for QC demonstration, with the only guideline the assumption that the target analyses would be performed after spatial normalization to an MNI anatomical template and not include the cerebellum.

Given such minimal requirements, I chose to preprocess the images with fMRIPrep 21.0.1 (Esteban et al., 2019; RRID:SCR_016216), which is reliable and straightforward to use, and has become our (and many other) group's default choice for fMRI preprocessing. Since surface analysis was not required, I chose to run fMRIPrep with volumetric preprocessing only, using the target MNI152NLin2009cAsym output template; the commands and generated text describing the preprocessing it performed are in the Supplementary material. No other preprocessing was done before the image QC procedures described in this manuscript.

### 2.2. Resources

Two documents were prepared for QC assessment: one for the fMRI (openQC_fMRIQC), and the other for the stimuli and behavioral performance (openQC_behav). Both the compiled (.pdf) and source (.rnw) versions of each are available at https://osf.io/ht543/. These are dynamic report files, written in R (version 3.6.3, RRID:SCR_001905; R Development Core Team, 2020) and knitr (version 1.39; Xie, 2014, 2015, 2022); all code is contained within the source (.rnw) versions of each file. The documents depend upon the RNifti (version 1.3.0; Clayden et al., 2020) and fields (version 11.6; Nychka et al., 2017) R packages, as well as AFNI 22.0.11 (RRID:SCR_005927; Cox, 1996; Cox and Hyde, 1997).

The task timing and responses in openQC_behav were read directly from the provided _events.tsv files. Similarly, openQC_fMRIQC read the six motion regressors and framewise displacement (FD) directly from the _desc-confounds_timeseries.tsv files produced by fMRIPrep (columns trans_x, trans_y, trans_z, rot_x, rot_y, rot_z, and framewise_displacement). The voxelwise temporal mean, standard deviation (sd), and tSNR (mean/sd) images were calculated with AFNI 3dTstat and 3dcalc functions, using the entire run (without censoring); see the startup code chunk in openQC_fMRIQC.rnw. While the number of censored frames (with FD above threshold) is included in the QC criteria as detailed below, I prefer not to censor when calculating the temporal mean, sd, and tSNR images during QC, to visually exaggerate differences between runs.

### 2.3. QC criteria

Four criteria necessary for task fMRI QC are presented below and applied to the FMRI Open QC Project task dataset. I want to emphasize that these are (in my opinion) necessary criteria, but not sufficient for all cases, nor a comprehensive list of all aspects of dataset quality. Indeed, while preparing this manuscript a reviewer commented that no criteria involved checking the raw (before preprocessing) anatomical or functional images. In our ongoing projects such a criterion is actually used: the experimenter rates the quality of the anatomical images immediately after acquisition, so that poor-quality scans can be repeated (https://osf.io/a7w39/). We no longer routinely evaluate the raw functional images, since when we have performed such checks they seem to add time and complexity without identifying issues beyond than those also found with the preprocessed images (Criterion D below). This decision to focus QC on preprocessed images is a judgement made for our particular research aims and resource limitations; please consider what is most important in your situation.

#### 2.3.1. Criterion A: Excessive motion

It can be surprisingly difficult to quantify how much motion is “excessive,” especially in fMRI datasets with high apparent motion (Inglis, 2016; Etzel, 2017a; Power et al., 2019; Fair et al., 2020). For task fMRI we have adapted the procedure described in Siegel et al. (2014), and censor individual frames with FD > 0.9. Further, if more than 20% of the frames in a run are censored, then the entire run is omitted (Etzel, 2017b; Etzel et al., 2022). While these are quantitative thresholds, I advise also viewing plots of the motion regressors during QC (first part of openQC_fMRIQC.pdf), and not solely rely on a count of censored frames or other summary statistic, since respiratory task entrainment, forceful blinking, machine vibrations, and many other things can cause unexpected (and potentially problematic) patterns in the motion regressors. We do not generally exclude runs or participants for an unusual motion pattern alone, but such patterns should be monitored as part of routine QC, since they may indicate that a problem is developing with image acquisition (e.g., a hardware fault), or help inform analysis strategy (e.g., if have respiratory task entrainment, including many motion regressors in the GLMs may remove substantial task information).

The censoring threshold of FD > 0.9 suggested here is much more lenient than advised by many researchers (including Siegel et al., 2014, which suggests 0.5 for typical adults), though we have found it a useful starting point. The choice of censoring threshold and method (e.g., on FD, enorm, or translation; single frame or adjacent as well) depends on multiple factors, perhaps most importantly study design and planned analysis. If temporal correlations will be used (e.g., for functional connectivity analyses), stringent motion thresholds and filtering techniques are essential (Satterthwaite et al., 2013; Ciric et al., 2017, 2018). With task designs, higher motion levels may be tolerable, if not strongly linked to trial types. The linkage of (apparent) motion and trial timing is common (Perl et al., 2019), however, and poses a serious methodological challenge. Plotting trial onsets with the motion regressors (as in openQC_fMRIQC.pdf) can aid in spotting potentially significant confounding of task and motion, but much work remains to be done in this area.

#### 2.3.2. Criterion B: Improper task presentation

To estimate task-related responses consistently across participants we generally need approximately the same amount of imaging data from each participant, so this criterion is to exclude a participant if < ½ of their trials have usable data (in the sense of being analyzable). Given the wide variety of task paradigms, the definition of “usable” data also varies, but at minimum both the fMRI images and task presentation details (e.g., stimulus onset time) must be present for the trials to be usable. For examples of the types of cases that may lead to this criterion being met, consider that incomplete task runs may result from hardware failure (e.g., projector bulb breaks; the participant mentions after scanning that they did not hear the audio stimuli), participant request (e.g., they ask to end a run early), or presentation error (e.g., the experimenter started the wrong task script; the task was programmed incorrectly and did not present the necessary trials).

While not implemented here, fMRI images for the run being present is not sufficient for a particular trial to be analyzable: it may have occurred during a period of excessive motion, and so be censored (which removes the affected frames from analysis). There is accordingly an interaction between criteria A and B: if a participant has many frames above the censoring threshold, the timing between the task trials and censored frames should be evaluated, as not all frames have the same impact. For example, some participants tend to move outside of task blocks (e.g., at the end of a run), which will change the number of analyzable trials less than if the motion occurs during the trials themselves.

#### 2.3.3. Criterion C: Invalid task performance

Note that this is not excluding participants who performed the task “incorrectly” according to the experimental hypotheses, but rather those who did not perform the expected task at all, such as not following the instructions or attending to the stimuli. For example, if the task involves responding to visual stimuli, we want to exclude participants who fell asleep or closed their eyes throughout stimulus presentation. Given the wide variety of protocols and priorities there is no universally applicable way to describe valid task performance; the most important aspects of each experiment should be considered, and criteria incorporate features like catch trials or eye gaze if present.

In the FMRI Open QC Project task dataset we only have the task information that can be gleaned from the BIDS events.tsv files; far less than is typical. Proceeding nevertheless, it appears that participants were asked to make a button-press response after every trial, the trials were fairly short and rapid (seven or more each minute; openQC_behav.pdf), and most participants responded accurately to most trials. In these types of designs it can work well to define invalid task performance quantitatively by no-response trials: exclude if a participant fails to respond to five or more trials in a row or more than 40 percent of the total trials within a run [thresholds adopted from the HCP task protocols (WU-Minn Consortium of the NIH Human Connectome Project, 2013)]. Note that this criterion is not of correct trial responses, but of any trial response (in cases where every trial requires a response).

Qualitatively, the responses should be reviewed for unambiguous patterns indicating that the participant was not performing the task correctly, such as using only one response button or responding in a repetitive sequence instead of to the stimuli.

The motivation for including quantitative thresholds is the need to distinguish inattention from poor task performance in the most unbiased way possible. Assuming the experimenters wish to include participants with a range of performance, people finding the task difficult will generally have a mix of correct- and incorrect-response trials, and slower RTs than people finding it easy. If the task requires a response to be made within a certain amount of time, slower RTs can lead to some trials not have a recorded response, even though the person was attentive and trying to perform the task. Thus, we may interpret 10 no-response trials differently if they were scattered evenly throughout the run (suggesting task difficulty, particularly if accompanied by low accuracy or slow RTs) than in a group of sequential trials (suggesting inattentiveness, particularly if trials with responses tend to be fast and accurate).

Plots such as in **Figure 3** and careful examination of response patterns in pilot data or previous experiments may assist in setting the quantitative thresholds for a particular experiment. The appropriateness of the quantitative thresholds of five or more no-response trials in a row or 40% of the total can't be evaluated in this case (given the lack of experimental details), but can serve as a default or starting point. While any threshold is imperfect, this may pose a smaller risk than that of experimenter bias if only qualitative criteria are used to determine which participants to exclude.

#### 2.3.4. Criterion D: Failed image acquisition and/or preprocessing

For this criterion, qualitatively review temporal mean, sd, and tSNR (mean/sd) images of each run, looking for incorrect or unusual cases requiring further investigation. Visual arrays with multiple runs side-by-side assist in evaluating typical variability, and thus also in spotting exceptions. We have found it useful to concentrate the initial QC evaluation on a few easy-to-spot features. First, check the volumetric temporal mean images for “alien” or “escaping” brains. No preprocessed image will exactly match the anatomic template, but distortions should not be extreme (“aliens”), and the brain should always be centered in the same part of the image (not “escaping” the frame). Second, the mean volumetric images should have clearly visible brain structure (i.e., resemble an anatomical scan), while the sd images should be brightest around the edges and in large vessels. Throughout, the images should be examined for unusually structured noise, dark areas, or other oddities. Surface images are more difficult to qualitatively review, since they are typically plotted on a single surface underlay and only include the gray matter ribbon. However, a useful QC feature is to look for the central sulcus in the temporal mean images, which should be clearly visible as “tiger stripes” at the top of each hemisphere; non-anatomical dark patches or “polka dot” patterns should also be noted.

If something is spotted during these qualitative checks of the statistical images, the run should be investigated in detail before deciding whether or not to exclude it. For example, if the raw (unprocessed) images appear as expected but the preprocessed images do not, an error likely occurred during preprocessing and may be possible to correct. If the raw images are also affected, then the run is likely unusable, and the source of the problem should be investigated to see if its recurrence can be avoided. Sometimes it is unclear whether an unusual run should be included or not, such as when dropout or noise is only slightly higher than typical. In these uncertain cases it can be useful to evaluate whether the results of positive control analyses (e.g., of strong effects such as button presses; Niso et al., 2022) are within the range of other participants, and exclude if not.

## 3. Results

Applying these criteria to the Open QC dataset, in my judgment three participants should be excluded from the hypothetical analysis: one for failed image acquisition (Table 1 criterion D and sub-010), and two for invalid task performance (Table 1 criterion C, sub-016, and sub-025). The others vary in quality, particularly of the images, but do not reach the exclusion thresholds. openQC_fMRIQC.pdf and openQC_behav.pdf (https://osf.io/ht543/) contain the necessary figures and statistics to evaluate the Open QC task dataset in terms of the Table 1 criteria, as will be described here.

**Table 1:** Task-based fMRI QC criteria: exclude the run for a subject if:

	**Name**	**Type**	**Details**
A	Excessive motion	Quantitative	20% or more of the frames have more than 0.9 mm FD
B	Improper task presentation	Quantitative	Less than half of the trials have usable data (e.g., due to hardware failure).
C	Invalid task performance (e.g., participant fell asleep)	Quantitative and qualitative	There is no response for five or more trials in a row or more than 40% of the total. Also exclude if the pattern of responses indicates unambiguously invalid performance (e.g., only one response button used).
D	Failed image acquisition and/or preprocessing	Qualitative	The preprocessed temporal mean, sd, and tSNR images do not resemble the MNI anatomical template (e.g., distorted shape), have the expected properties (e.g., the sd image does not resemble an arteriogram), have unusual structured noise, or are otherwise clearly and excessively affected by artifacts.

### 3.1. Results from applying the image-related criteria

The first document, openQC_fMRIQC.pdf, is intended to highlight key image-related features of the task fMRI runs: motion (criterion A) and success of acquisition and preprocessing (criterion D). The first section has line plots of the six realignment parameters and FD for each run, with trial onsets, censoring threshold (FD > 0.9), and censored frames marked. The number and proportion of censored frames are printed on each plot, for ease of comparing to the 0.2 censoring exclusion criterion. No participants reached this threshold, and while movement clearly varies across participants, I do not suggest excluding any due to excessive (or highly unusual) movement. Interestingly, the degree of apparent motion varies across participants; for example minimal in sub-011 and sub-030, but clear in sub-012 and sub-022. Some participants have brief instances of overt head motion, such as sub-003 and sub-018. Overall, the FD > 0.9 frame censoring threshold seems reasonable for this dataset, appropriately identifying the larger overt, but not apparent, head motion.

**Figure 1:**
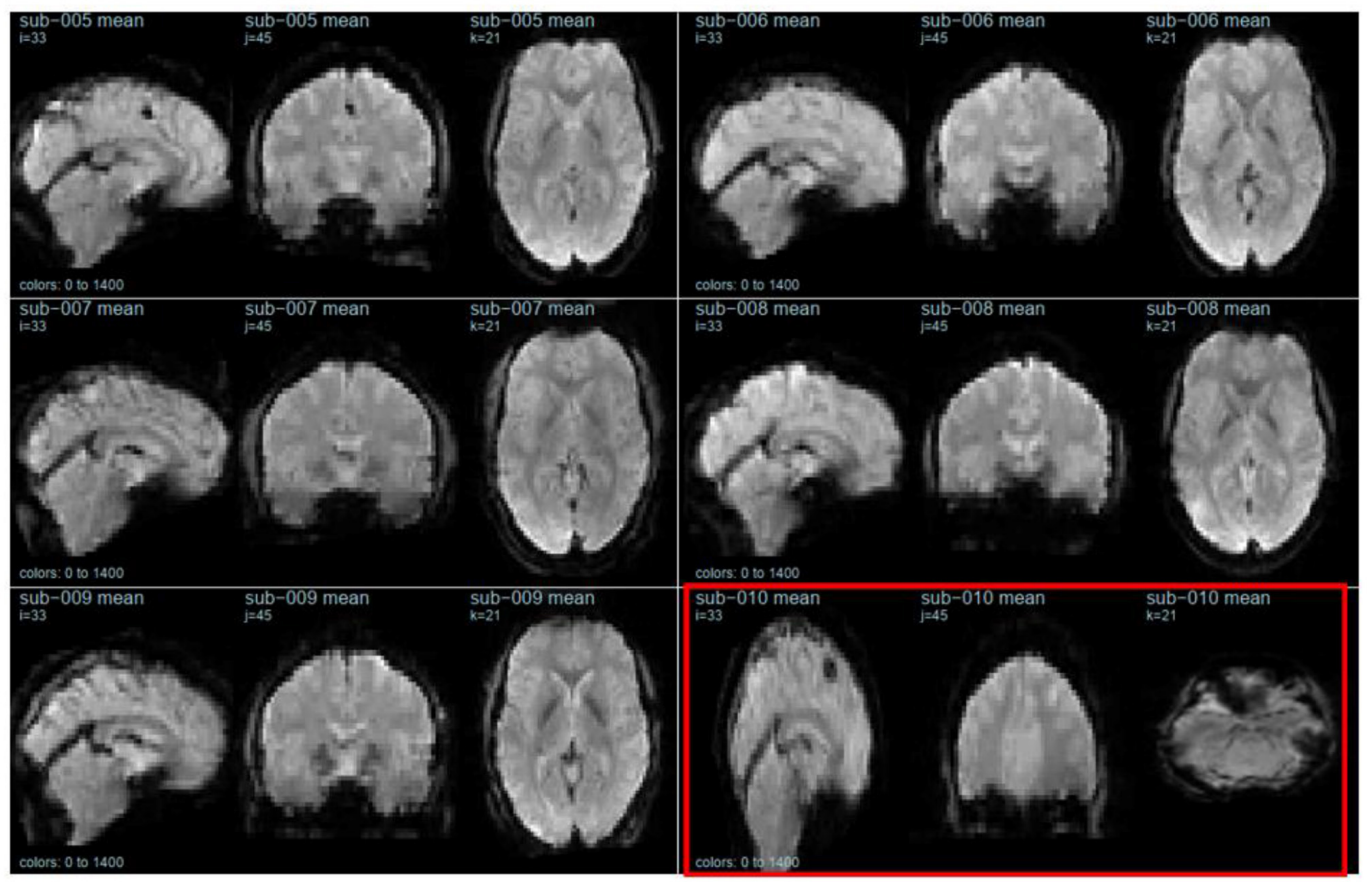
Temporal mean images for six participants, calculated after preprocessing. sub-010 (outlined in red) is highly distorted. This figure is from page 12 of openQC_fMRIQC.pdf.

**Figure 2:**
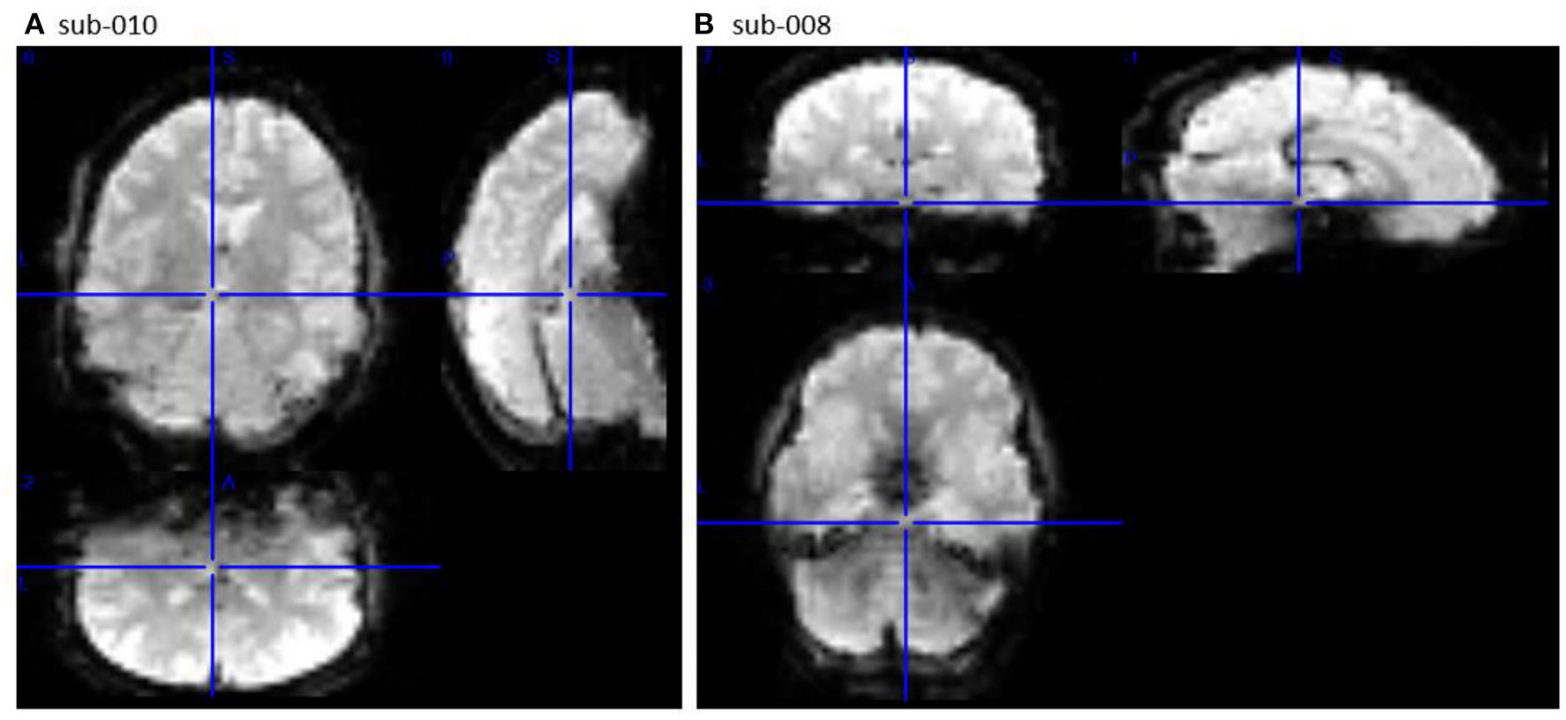
Frame 100 of the raw image timeseries (_bold.nii.gz) for sub-010 [left, **(A)**] and comparison sub-008 [right, **(B)**], viewed in MRIcron (Rorden et al., 2007; RRID:SCR_002403). sub-010 was not acquired with the same parameters as sub-008 (and the other participants).

The second section of openQC_fMRIQC.pdf has plots of the temporal mean, standard deviation, and tSNR (mean/sd) images for each participant, for applying criterion D (or more concretely, looking for oddities; images that are not like the others). As shown in Figure 1, while all other participants' images resemble the MNI preprocessing target template, sub-010 is clearly a different shape. Other than the distorted sub-010 images, the summary images have the expected characteristics (e.g., anatomical structures are visible on the means; sd have bright vessels). To further evaluate sub-010, I looked at the provided raw image file (sub-010_task-pamenc_bold.nii.gz); if the raw image looks typical, we could suspect that the problem was introduced during preprocessing. However, here, the problem is present in the raw image as well: Figure 2 shows frame 100 from sub-010 on the left, and for a comparison example, sub-008 on the right. The image orientation and planes are clearly different for sub-010, so the unusual appearance in Figure 1 was not introduced by preprocessing. Further, the phase encoding direction and other fields in sub-010_task-pamenc_bold.json vary from the other participants. We can thus conclude that the image acquisition was incorrect for sub-010, and the participant's imaging data should be excluded.

In some cases the raw images first appear odd, but are recoverable (e.g., by changing parameters or preprocessing template). Other issues arise from errors that causes the images to have fundamentally different properties than the rest of the dataset (e.g., if the wrong head coil or encoding direction was used), and so must be excluded. If this was an ongoing experiment, the researchers should investigate how it came about that the wrong acquisition protocol was used for the session, and if changes to the SOPs [Standard Operating Procedures (Etzel et al., 2022; Niso et al., 2022), https://osf.io/6r9f8] could avoid the mistake happening again.

### 3.2. Results from applying the task-related criteria

The second document, openQC_behav.pdf, is intended to highlight and evaluate key aspects of the task presentation (criterion B) and behavioral performance (criterion C). The code in chunk code2 counts how many trials of each type were presented to each participant, and prints an error message if the counts are not as expected. For this dataset, it checks the number of CONTROL and TASK trials in each run, and since all participants have the same number, no participants were excluded for criterion B. If some aspect of the task paradigm is key for valid analysis (e.g., each stimulus must be presented exactly twice), this should be explicitly tested in this section, and any violations clearly highlighted.

**Figure 3:**
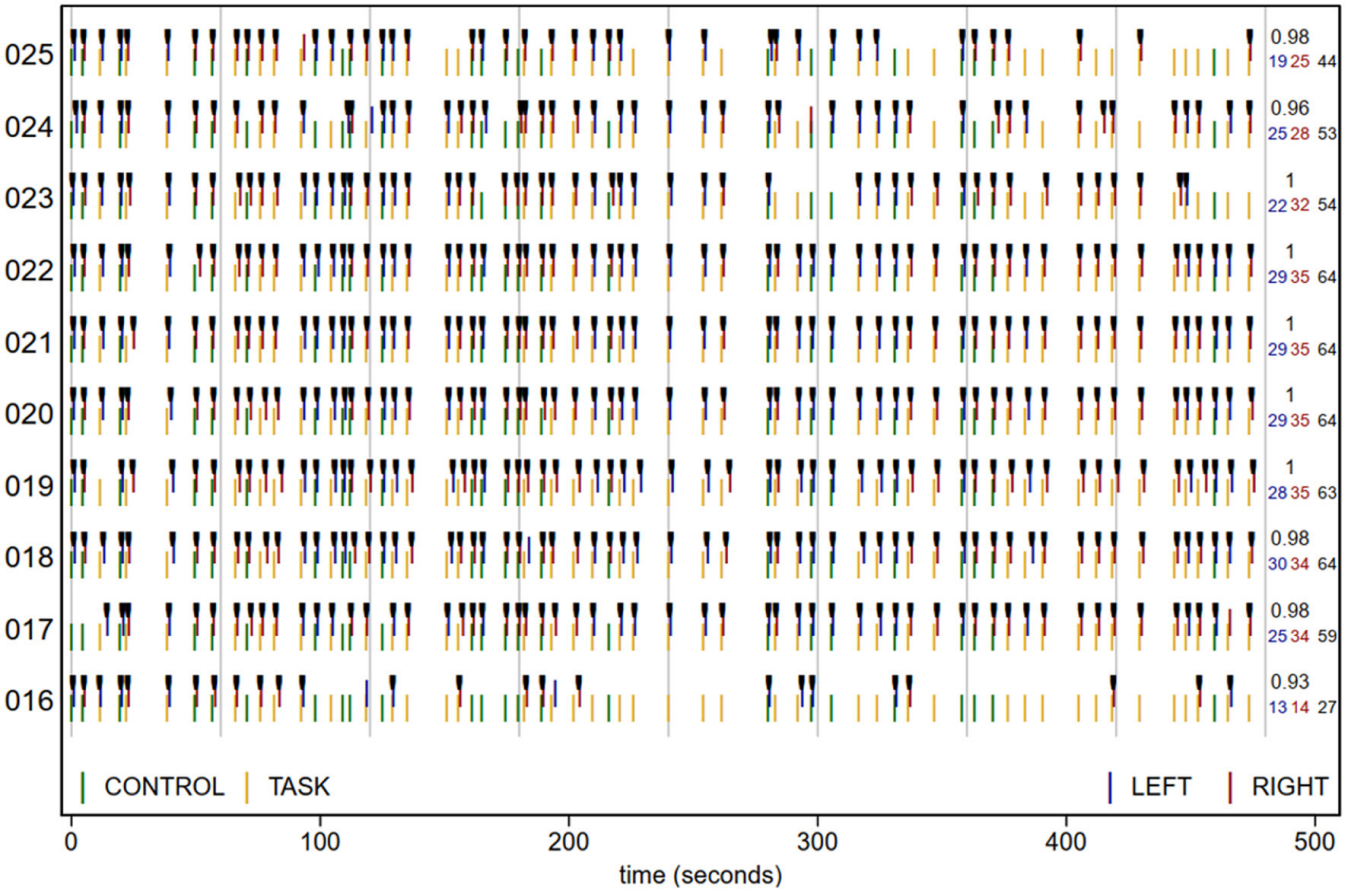
Task trial onset time (colored by trial type), response (at onset + reaction time), and accuracy (black ticks) for 10 participants. Vertical gray lines are at 1-min intervals. Numbers at right margin give the number of LEFT (blue) and RIGHT (red) responses, total responses (black; 64 if no trials lacked a response), and proportion correct of the trials with a response. sub-016 responded correctly to all trials in the 1st min, but then had more and more trials without a response, suggesting that they became less attentive as the run progressed. sub-025 was also excluded due to criterion C, and while their strings of no-response trials did not reach the 5-trial threshold until the last minute of the run, they missed noticeably more trials in the second than first half of the run.

The plot in openQC_behav.pdf, excerpted in Figure 3, summarizes the task presentation and performance for each participant (y axis). Time is along the x axis, and each green (CONTROL) and yellow (TASK) plotting symbol indicates the type and onset time of a trial (read from the origcopy _events.tsv files). The blue (LEFT) and red (RIGHT) lines show the time and type of each button press, with black tick marks on correct responses. The numbers in the right margin list the number of LEFT and RIGHT responses, their total, and the proportion correct of trials with a response. While dense, with practice a lot of task and performance information can be quickly scanned in plots like these, including trial timing and randomization (e.g., here we can see that all participants had the same trial order and timing), and unexpected response patterns.

To reduce the chances of missing an exception, the quantitative task performance criteria (C) are tested explicitly in chunk code2. Three notifications are printed: that sub-016 has both >40% no-response trials and 5 or more no-response trials in a row, and that sub-025 has 5 or more no-response trials in a row. These strings of trials without a response are visible in the participants' rows in Figure 3, as stretches of trial onsets (green and yellow lines) without the corresponding responses (red and blue lines). Accordingly, both sub-016 and sub-025 should be excluded from analysis due to excessive missing responses. We can also observe that participants made very few errors in this experiment; nearly all responses that were made, were correct (sub-013 is lowest at 0.88 accuracy). In some paradigms or analyses it may be relevant to establish additional criteria, such as excluding participants with accuracy below a threshold.

## 4. Discussion

This article presented an evaluation of the FMRI Open QC Project task dataset, as part of the Demonstrating Quality Control Procedures in fMRI methodological research project. Both the task and fMRI aspects of the dataset were examined, applying the criteria summarized in Table 1 *via* the figures and statistics in the two dynamic report summary documents (openQC_fMRIQC.pdf and openQC_behav.pdf; R, AFNI, and LaTeX) available at https://osf.io/ht543/. Using these criteria, I suggest that three participants should be excluded from the hypothetical analysis: one for failed image acquisition and two for invalid task performance.

I do not believe that there is a “perfect” or even “ideal” procedure for QC in psychological or neuroimaging research: new potential issues are identified continually, and the sheer amount of data makes checking every piece impossible. Nevertheless, there clearly is a terrible way to do QC: by omission. We have likely all been involved in a project where a critical artifact or error was discovered late, sometimes so severe that the dataset must be abandoned or a publication corrected.

Since the FMRI Open QC Project task dataset was complete (acquisition finished years ago) and small (only one run per person), I included all of the participants in each of the two QC summary documents. This is only appropriate on completed datasets, however. For new and ongoing projects, QC summary documents should be created for each participant on a continual basis, and reviewed as soon after each session as possible, a task made efficient by dynamic reports and clear guidance on how to review the reports [examples of such single-subject QC reports from the DMCC project (Braver et al., 2021; Etzel et al., 2022) are at https://osf.io/7xkq9 and https://osf.io/z62s5]. While no one can guarantee that such ongoing QC procedures will prevent disaster, they can certainly help reduce the odds of collecting an unusable dataset, by allowing researchers to catch serious issues early, when they can still be corrected.

The material presented here is intended to serve both as inspiration and a template for adapting to your own datasets. The code in the summary documents is designed to be straightforward and approachable; easy to edit for other datasets or reimplement in a new language. A number of QC software packages which can generate reports without programming are also available, including MRIQC (Esteban et al., 2017). However, accomplished, I suggest QC include reviewing the images themselves, not only summary statistics.

Particularly with task fMRI, but to some extent with any study of living participants, both the task/behavior and imaging parts of the dataset need to be included in the QC. Which aspects and criteria are most important will vary with each dataset and analysis, so I suggest starting by considering which features absolutely must be true for the analyses and inferences to be valid, and ensuring that those features are covered in the QC procedures. We may not be able to achieve “perfect” QC, analysis, or results, but good QC procedures can let us be confident that what we are analyzing and reporting is real, not wholly invalid.

## Data availability statement

Publicly available datasets were analyzed in this study. This data can be found at: https://osf.io/qaesm/.

## Author contributions

JE wrote the manuscript and code and conducted the analyses.
