# Supplementary material for: Efficient evaluation of the Open QC task fMRI dataset
Source: Front Neuroimaging. 2023 Feb 17;2:1070274. doi: 10.3389/fnimg.2023.1070274 (PMC10406291; doi:10.3389/fnimg.2023.1070274)
Supplement: Supplementary file 1 [file Data_Sheet_1.docx]

Supplementary Material

# Supplementary Files

openQC_behav.pdf (compiled) and openQC_behav.rnw (source)

openQC_fMRIQC.pdf (compiled) and openQC_fMRIQC.rnw (source)

# Supplementary Figures and Tables

Table S1. Excluded/uncertain subjects in the OpenQC task dataset.

| **SUB ID** | **EXCLUDE** | **UNCER.** | **COMMENT** |
| --- | --- | --- | --- |
| sub-010 | X |  | D: Incorrect image acquisition parameters. |
| sub-016 | X |  | C: Too many trials without a response (>40% total; 5 or more in a row). |
| sub-025 | X |  | C: Too many trials without a response (5 or more in a row). |

# Supplementary Text

## Bash script for running fMRIPrep

Bash commands and settings used to run fMRIPrep 21.0.1 for sub-023 of the FMRI Open QC Project task dataset.

SUBID=023

LOGGINGTIMESTAMP() { date -Ins; }

CURRENTCPUUSE() { echo $[100-$(vmstat 1 2|tail -1|awk '{print $15}')]; }

FREESPACEINCURRENTDIR() { printf '%s\n' $(df -BG -P . | tail -1 | awk '{print $4}' | sed s'/G$//'); }

PRIMARY_WORKING_DIR="/scratch2/JoEtzel/openQC/"

pushd ${PRIMARY_WORKING_DIR}

mkdir -p ${PRIMARY_WORKING_DIR}${SUBID}/.license/freesurfer/

cp /scratch2/license.txt ${PRIMARY_WORKING_DIR}${SUBID}/.license/freesurfer/license.txt

/opt/singularity/bin/singularity run --cleanenv -B \

${PRIMARY_WORKING_DIR}${SUBID}:/tmp \

/data/nil-bluearc/ccp-hcp/SingularityImages/fmriprep-21.0.1.simg \

--fs-license-file /tmp/.license/freesurfer/license.txt \

-w /tmp /tmp/BIDS /tmp/derivatives \

participant --participant_label ${SUBID} \

--output-spaces fsaverage5 MNI152NLin2009cAsym \

--n-cpus 16 \

--omp-nthreads 4 \

--mem-mb 64000 -v \

--fs-no-reconall >> ${PRIMARY_WORKING_DIR}${SUBID}/${SUBID}_fmriprep_singularity_sose.txt 2>&1

## Description of fMRIPrep preprocessing

To avoid confusion, the section describing several statistics (annotated motion outliers, DVARS, and CompCor) not used in the current analysis was removed from the boilerplate text.

Results included in this manuscript come from preprocessing performed using fMRIPrep 21.0.1 (Esteban et al. 2019; 2018) (RRID:SCR_016216), which is based on Nipype 1.6.1 (Gorgolewski et al. 2018; 2011) (RRID:SCR_002502).

**Anatomical data preprocessing**

One T1-weighted (T1w) image was found within the input BIDS dataset for each subject. The T1-weighted (T1w) image was corrected for intensity non-uniformity (INU) with N4BiasFieldCorrection (Tustison et al. 2010), distributed with ANTs 2.3.3 (Avants et al. 2008) (RRID:SCR_004757), and used as T1w-reference throughout the workflow. The T1w-reference was then skull-stripped with a Nipype implementation of the antsBrainExtraction.sh workflow (from ANTs), using OASIS30ANTs as target template. Brain tissue segmentation of cerebrospinal fluid (CSF), white-matter (WM) and gray-matter (GM) was performed on the brain-extracted T1w using fast (FSL 6.0.5.1:57b01774, RRID:SCR_002823) (Zhang, Brady, and Smith 2001). Volume-based spatial normalization to one standard space (MNI152NLin2009cAsym) was performed through nonlinear registration with antsRegistration (ANTs 2.3.3), using brain-extracted versions of both T1w reference and the T1w template. The following template was selected for spatial normalization: ICBM 152 Nonlinear Asymmetrical template version 2009c [RRID:SCR_008796; TemplateFlow ID: MNI152NLin2009cAsym] (Fonov et al. 2009).

**Functional data preprocessing**

For each subject’s BOLD run the following preprocessing was performed. First, a reference volume and its skull-stripped version were generated using a custom methodology of fMRIPrep. Head-motion parameters with respect to the BOLD reference (transformation matrices, and six corresponding rotation and translation parameters) are estimated before any spatiotemporal filtering using mcflirt (FSL 6.0.5.1:57b01774) (Jenkinson et al. 2002). BOLD runs were slice-time corrected to 0.974s (0.5 of slice acquisition range 0s-1.95s) using 3dTshift from AFNI (RRID:SCR_005927) (Cox and Hyde 1997). The BOLD time-series (including slice-timing correction when applied) were resampled onto their original, native space by applying the transforms to correct for head-motion. These resampled BOLD time-series will be referred to as preprocessed BOLD in original space, or just preprocessed BOLD. The BOLD reference was then co-registered to the T1w reference using mri_coreg (FreeSurfer) followed by flirt (FSL 6.0.5.1:57b01774)(Jenkinson and Smith 2001) with the boundary-based registration (Greve and Fischl 2009) cost-function. Co-registration was configured with six degrees of freedom. Several confounding time-series were calculated based on the preprocessed BOLD: framewise displacement (FD), DVARS and three region-wise global signals. FD was computed using two formulations following Power (absolute sum of relative motions (Power et al. 2014)) and Jenkinson (relative root mean square displacement between affines (Jenkinson et al. 2002)). FD and DVARS are calculated for each functional run, both using their implementations in Nipype (following the definitions by (Power et al. 2014)).

The BOLD time-series were resampled into standard space, generating a preprocessed BOLD run in MNI152NLin2009cAsym space. First, a reference volume and its skull-stripped version were generated using a custom methodology of fMRIPrep. All resamplings can be performed with a single interpolation step by composing all the pertinent transformations (i.e., head-motion transform matrices, susceptibility distortion correction when available, and co-registrations to anatomical and output spaces). Gridded (volumetric) resamplings were performed using antsApplyTransforms (ANTs), configured with Lanczos interpolation to minimize the smoothing effects of other kernels (Lanczos 1964).

Many internal operations of fMRIPrep use Nilearn 0.8.1 (RRID:SCR_001362) (Abraham et al. 2014), mostly within the functional processing workflow. For more details of the pipeline, see the section corresponding to workflows in fMRIPrep’s documentation (https://fmriprep.readthedocs.io/en/latest/workflows.html).

# References

Abraham, Alexandre, Fabian Pedregosa, Michael Eickenberg, Philippe Gervais, Andreas Mueller, Jean Kossaifi, Alexandre Gramfort, Bertrand Thirion, and Gael Varoquaux. 2014. “Machine Learning for Neuroimaging with Scikit-Learn.” *Frontiers in Neuroinformatics* 8. https://doi.org/10.3389/fninf.2014.00014.

Avants, B.B., C.L. Epstein, M. Grossman, and J.C. Gee. 2008. “Symmetric Diffeomorphic Image Registration with Cross-Correlation: Evaluating Automated Labeling of Elderly and Neurodegenerative Brain.” *Medical Image Analysis* 12 (1): 26–41. https://doi.org/10.1016/j.media.2007.06.004.

Cox, Robert W., and James S. Hyde. 1997. “Software Tools for Analysis and Visualization of FMRI Data.” *NMR in Biomedicine* 10 (4–5): 171–78. https://doi.org/10.1002/(SICI)1099-1492(199706/08)10:4/5<171::AID-NBM453>3.0.CO;2-L.

Esteban, Oscar, Ross Blair, Christopher J. Markiewicz, Shoshana L. Berleant, Craig Moodie, Feilong Ma, Ayse Ilkay Isik, et al. 2018. “FMRIPrep.” *Software*. https://doi.org/10.5281/zenodo.852659.

Esteban, Oscar, Christopher J. Markiewicz, Ross W. Blair, Craig A. Moodie, A. Ilkay Isik, Asier Erramuzpe, James D. Kent, et al. 2019. “FMRIPrep: A Robust Preprocessing Pipeline for Functional MRI.” *Nature Methods* 16 (1): 111–16. https://doi.org/10.1038/s41592-018-0235-4.

Fonov, VS, AC Evans, RC McKinstry, CR Almli, and DL Collins. 2009. “Unbiased Nonlinear Average Age-Appropriate Brain Templates from Birth to Adulthood.” *NeuroImage* 47, Supplement 1: S102. https://doi.org/10.1016/S1053-8119(09)70884-5.

Gorgolewski, Krzysztof J., C. D. Burns, C. Madison, D. Clark, Y. O. Halchenko, M. L. Waskom, and S. Ghosh. 2011. “Nipype: A Flexible, Lightweight and Extensible Neuroimaging Data Processing Framework in Python.” *Frontiers in Neuroinformatics* 5: 13. https://doi.org/10.3389/fninf.2011.00013.

Gorgolewski, Krzysztof J., Oscar Esteban, Christopher J. Markiewicz, Erik Ziegler, David Gage Ellis, Michael Philipp Notter, Dorota Jarecka, et al. 2018. “Nipype.” Zenodo. https://doi.org/10.5281/zenodo.596855.

Greve, Douglas N, and Bruce Fischl. 2009. “Accurate and Robust Brain Image Alignment Using Boundary-Based Registration.” *NeuroImage* 48 (1): 63–72. https://doi.org/10.1016/j.neuroimage.2009.06.060.

Jenkinson, Mark, Peter Bannister, Michael Brady, and Stephen Smith. 2002. “Improved Optimization for the Robust and Accurate Linear Registration and Motion Correction of Brain Images.” *NeuroImage* 17 (2): 825–41. https://doi.org/10.1006/nimg.2002.1132.

Jenkinson, Mark, and Stephen Smith. 2001. “A Global Optimisation Method for Robust Affine Registration of Brain Images.” *Medical Image Analysis* 5 (2): 143–56. https://doi.org/10.1016/S1361-8415(01)00036-6.

Lanczos, C. 1964. “Evaluation of Noisy Data.” *Journal of the Society for Industrial and Applied Mathematics Series B Numerical Analysis* 1 (1): 76–85. https://doi.org/10.1137/0701007.

Power, Jonathan D., Anish Mitra, Timothy O. Laumann, Abraham Z. Snyder, Bradley L. Schlaggar, and Steven E. Petersen. 2014. “Methods to Detect, Characterize, and Remove Motion Artifact in Resting State FMRI.” *NeuroImage* 84 (Supplement C): 320–41. https://doi.org/10.1016/j.neuroimage.2013.08.048.

Tustison, N. J., B. B. Avants, P. A. Cook, Y. Zheng, A. Egan, P. A. Yushkevich, and J. C. Gee. 2010. “N4ITK: Improved N3 Bias Correction.” *IEEE Transactions on Medical Imaging* 29 (6): 1310–20. https://doi.org/10.1109/TMI.2010.2046908.

Zhang, Y., M. Brady, and S. Smith. 2001. “Segmentation of Brain MR Images through a Hidden Markov Random Field Model and the Expectation-Maximization Algorithm.” *IEEE Transactions on Medical Imaging* 20 (1): 45–57. https://doi.org/10.1109/42.906424.
